# Supplementary material for: Sensitivity of RT-PCR testing of upper respiratory tract samples for SARS-CoV-2 in hospitalised patients: a retrospective cohort study
Source: Wellcome Open Res. 2022 Feb 1;5:254. Originally published 2020 Oct 26. [Version 2] doi: 10.12688/wellcomeopenres.16342.2 (PMC8815009; doi:10.12688/wellcomeopenres.16342.2)
Supplement: Peer review history

# Peer review: Reviewer response for version 2

**DOI:** 10.21956/wellcomeopenres.19487.r48427

**Published:** 2022-02-03

**Authors:** Mariska M.G. Leeflang

**Affiliations:** 1 Department of Epidemiology and Data Science, Amsterdam University Medical Centers, University of Amsterdam, Amsterdam, 1100 DE, The Netherlands

I have no further comments. They addressed most of my previous comments.

Is the work clearly and accurately presented and does it cite the current literature?

Partly

If applicable, is the statistical analysis and its interpretation appropriate?

Partly

Are all the source data underlying the results available to ensure full reproducibility?

No

Is the study design appropriate and is the work technically sound?

Partly

Are the conclusions drawn adequately supported by the results?

Yes

Are sufficient details of methods and analysis provided to allow replication by others?

Partly

Reviewer Expertise:

NA

I confirm that I have read this submission and believe that I have an appropriate level of expertise to confirm that it is of an acceptable scientific standard.

# Peer review: Reviewer response for version 1

**DOI:** 10.21956/wellcomeopenres.17968.r42151

**Published:** 2021-02-08

**Authors:** Mariska M.G. Leeflang

**Affiliations:** 1 Department of Epidemiology and Data Science, Amsterdam University Medical Centers, University of Amsterdam, Amsterdam, 1100 DE, The Netherlands

The sensitivity of RTT-PCR for COVID-19 or SARS-CoV-2 infection is an estimate of much debate, so any study providing more information on what the actual sensitivity (and specificity) should be is very much desired. Although I wonder whether this is the study that will really provide us with the right answers, the conclusion ( *“Reliance on RT-PCR testing may result in patients with COVID-19 being inappropriately labelled with alternative diagnoses.”*) will probably still be true.

1. Abstract, Results: *“The sensitivity of two upper respiratory tract RT-PCR tests increased sensitivity to 90.6%”*. Please rephrase. Suggestion: *“Using two upper respiratory tract RT-PCR tests increased sensitivity to 90.6%”*.
2. Introduction: *“Overestimation of sensitivity may lead to patients with disease being incorrectly diagnosed”*. I think it is not necessarily the overestimation leading to misdiagnosis, but rather the sensitivity (and actually the fact that a test results in any false negative) in itself.
3. The authors state that they comply with the STROBE guidelines for reporting, but as this is a diagnostic accuracy study, they should comply to STARD.
4. From the methods section it is not direct clear who are the tested population: only hospitalized patients, or also people in the community, or both? Please be clear.
5. The authors state that they ‘identified’ URT samples. What does this mean? Does this also mean that this is a retrospective study?
6. Only unambiguous results were used, but this leads to a high risk of bias and I think that at least the total number of ambiguous test results should have been reported. Also, what happened to samples from patients with one or more ambiguous test results. Were these patients excluded, or only the ambiguous samples? And how many of the patients with only one sample also had an ambiguous sample result?
7. Probably due to the retrospective nature, the assessment of the reference standard/gold standard was not blinded for the index test result (as the way the results were verified depended on whether there were one or more negative test results). This may have led to high risk of bias, especially when the index test (RT-PCR) was part of the clinical diagnosis criteria.
8. Please make it explicit whether the RT-PCR was part of the clinical diagnosis criteria.
9. Please make it explicit whether the outcomes and diagnoses were known when the RT-PCR results were assessed.
10. I was surprised that single negative test results were considered true negative and multiple negative test results were checked extra. To me, two or three negative test results would provide more certainty than only one negative test result.
11. Please check the estimates of sensitivity and specificity reported in the abstract and in the full text. It seems that they are different. Maybe they are not, but then it may be helpful to explain the numbers a bit more in the full text.
12. Also, I had difficulty in getting the numbers of tested patients right…Is it correct that over 5000 patients were not included in the study? That is the majority of the eligible patients. Why was this? And could that have led to biased results?
13. Title versus abstract: the title only mentions sensitivity, while the aim of the study (at least in the abstract) involves both sensitivity and specificity. Please be consistent in this. Also, if the aim of the study was to investigate specificity, why were potential false positives not further followed up? So maybe elucidating the specificity was not the aim of this study after all.
14. Limitations: the retrospective design and the lack of blinding should also be mentioned as limitations.
15. General remark: also minor detail. I think that using the abbreviation URT is not necessary and does not improve the readability of the manuscript.

Is the work clearly and accurately presented and does it cite the current literature?

Partly

If applicable, is the statistical analysis and its interpretation appropriate?

Partly

Are all the source data underlying the results available to ensure full reproducibility?

No

Is the study design appropriate and is the work technically sound?

Partly

Are the conclusions drawn adequately supported by the results?

Yes

Are sufficient details of methods and analysis provided to allow replication by others?

Partly

Reviewer Expertise:

Clinical epidemiology; co-author of QUADAS-2, the risk of bias tool for diagnostic accuracy studies.

I confirm that I have read this submission and believe that I have an appropriate level of expertise to confirm that it is of an acceptable scientific standard, however I have significant reservations, as outlined above.

# Peer review: Reviewer response for version 1

**DOI:** 10.21956/wellcomeopenres.17968.r41294

**Published:** 2020-11-19

**Authors:** James Otieno

**Affiliations:** 1 Department of Microbiology, Icahn School of Medicine at Mount Sinai, New York City, NY, USA; 2 Division of International Epidemiology and population Studies (DIEPS), Fogarty International Center, NIH, Bethesda, MD, USA

The coronavirus disease 2019 (COVID-19) pandemic is a serious global problem and proper diagnosis of individuals is a key step in the control strategy. As stated by the authors, a wrongful diagnosis could either send positive individuals into non-COVID-19 areas where they could be a risk of infection to others or send negative individuals into COVID-19 areas with a risk of infection unto themselves.

 Using a large dataset, the authors highlight the importance of (i) not relying on a single method for diagnosis as a gold standard, either clinical or RT-PCR alone, and (ii) RT-PCR repeat testing that markedly improves test sensitivity.

***Minor comment:***

 The single negative test classified as a true negative, were these cases that never met a clinical diagnosis of COVID-19 (hence not suspected as a false negative)? Or is it that no other samples were available/collected? Would the edit below bring more clarity?

*“Patients with a single negative test result were classified as a true negative **if they never met a clinical diagnosis of COVID-19**. The clinical guidelines in place at the time specified that if there was clinical suspicion of COVID-19, an URT RT-PCR test should be repeated if the first test was negative.”*

Is the work clearly and accurately presented and does it cite the current literature?

Yes

If applicable, is the statistical analysis and its interpretation appropriate?

Yes

Are all the source data underlying the results available to ensure full reproducibility?

Yes

Is the study design appropriate and is the work technically sound?

Yes

Are the conclusions drawn adequately supported by the results?

Yes

Are sufficient details of methods and analysis provided to allow replication by others?

Yes

Reviewer Expertise:

Molecular Epidemiology and Bioinformatics.

I confirm that I have read this submission and believe that I have an appropriate level of expertise to confirm that it is of an acceptable scientific standard.
